# A Narrative Review of Network Studies in Depression: What Different Methodological Approaches Tell Us About Depression

**DOI:** 10.3389/fpsyt.2021.719490

**Published:** 2021-10-28

**Authors:** Marieke Wichers, Harriëtte Riese, Taylor M. Hodges, Evelien Snippe, Fionneke M. Bos

**Affiliations:** ^1^University of Groningen, University Medical Center Groningen, Department of Psychiatry, Interdisciplinary Center Psychopathology and Emotion regulation, Groningen, Netherlands; ^2^University of Groningen, University Medical Center Groningen, Department of Psychiatry, Rob Giel Research Center, Groningen, Netherlands

**Keywords:** network theory, network analysis, major depressive disorder, experience sampling method (ESM)/ecological momentary assessment (EMA), review (article), momentary affect dynamics theory

## Abstract

The network theory of psychopathology proposes that mental disorders arise from direct interactions between symptoms. This theory provides a promising framework to understand the development and maintenance of mental disorders such as depression. In this narrative review, we summarize the literature on network studies in the field of depression. Four methodological network approaches are distinguished: (i) studies focusing on symptoms at the macro-level vs. (ii) on momentary states at the micro-level, and (iii) studies based on cross-sectional vs. (iv) time-series (dynamic) data. Fifty-six studies were identified. We found that different methodological approaches to network theory yielded largely inconsistent findings on depression. Centrality is a notable exception: the majority of studies identified either positive affect or anhedonia as central nodes. To aid future research in this field, we outline a novel complementary network theory, the momentary affect dynamics (MAD) network theory, to understand the development of depression. Furthermore, we provide directions for future research and discuss if and how networks might be used in clinical practice. We conclude that more empirical network studies are needed to determine whether the network theory of psychopathology can indeed enhance our understanding of the underlying structure of depression and advance clinical treatment.

## Highlights

- This narrative review evaluates empirical network studies on depression.
- We summarize findings regarding comorbidity, centrality, and network connectivity.
- Different network methodological approaches yield inconsistent findings.
- Important challenges for future network research are outlined.
- Empirical evidence is not yet suggestive of the use of networks as a clinical tool.

## Introduction

The network theory of psychopathology has gained increasing popularity in recent years (1, 2). This theory postulates that a psychiatric syndrome, such as depression, arises not because of the presence of a latent cause, but rather due to a process in which psychological states or symptoms trigger each other. The theory assumes that this process will eventually result in a cluster of co-occurring symptoms, which we call mental disorders. Empirical research regarding the application of the network theory and its techniques is exponentially growing (3). The popularity of the network theory is also expressed in the eagerness of healthcare professionals to apply these ideas within clinical practice (4–7). Thus, the field is in urgent need of a comprehensive evaluation of the findings of empirical network studies.

Such an evaluation should take into account that different methodological approaches have been used within each network study, each yielding their own interpretations and implications. These methodological approaches are different, but complementary, operationalizations of how network dynamics may explain the development and maintenance of psychopathology. Networks consists of nodes and edges (the connections between nodes). In networks on psychopathology, nodes have so far signified either clinical symptoms that supposedly operate at the macro-level (1), or momentary affective states operating at a much smaller timescale, termed the micro-level (8). Typically, the macro-level network approach is used to investigate between-person relationships among symptoms cross-sectionally at a given time point in a large group of individuals. The micro-level network approach, on the other hand, is often used to examine the processes underlying the development of clinical symptoms by studying the dynamics between everyday life momentary affective states.

**Figure 1:**
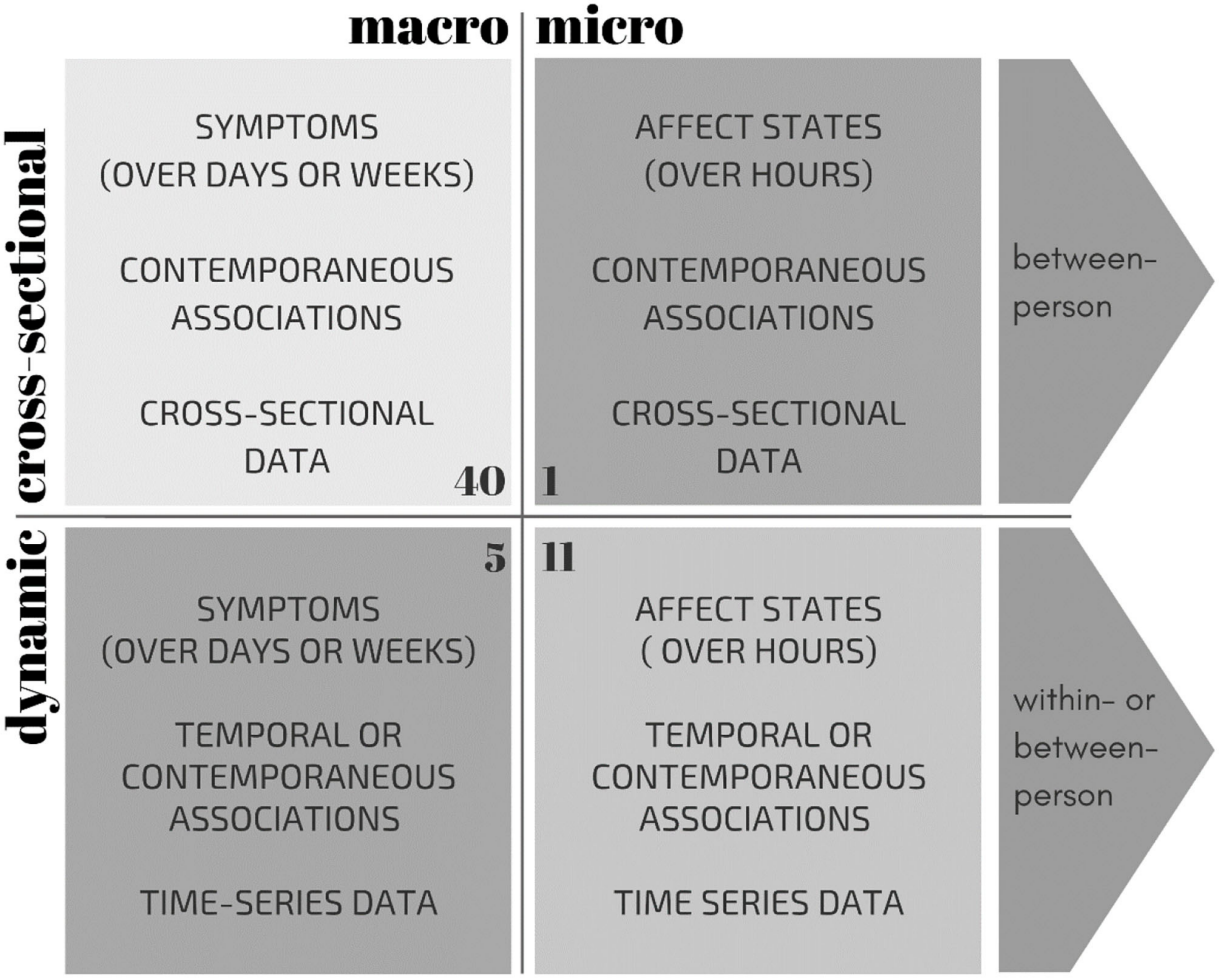
Overview of characteristics of the four methodological approaches to network theory: micro-level affective states vs. macro-level symptoms, based on cross-sectional vs. dynamic (time-series) data. The number of studies are indicated in the corner of each quadrant (total *N* = 56). Note that one study constructed both a cross-sectional and a dynamic network, and is therefore referenced twice in this Figure.

In addition to differences in macro- vs. micro-level approaches, network studies also differ in the type of data on which they base their network analyses, namely: cross-sectional (data with one assessment per participant) vs. time-series data (data with multiple assessments per participant). Often, cross-sectional network studies examine symptoms on the macro-level, whereas dynamic network studies use time-series data to examine momentary affect at the micro-level. Although it might be expected that the distinction between micro- and macro-level experiences is rather a continuum than distinct categories, for the purposes of this narrative review, we have placed each network study in one of four quadrants: cross-sectional^1^ vs. dynamic associations and micro- vs. macro-level approaches (see Figure 1).

**Figure 2:**
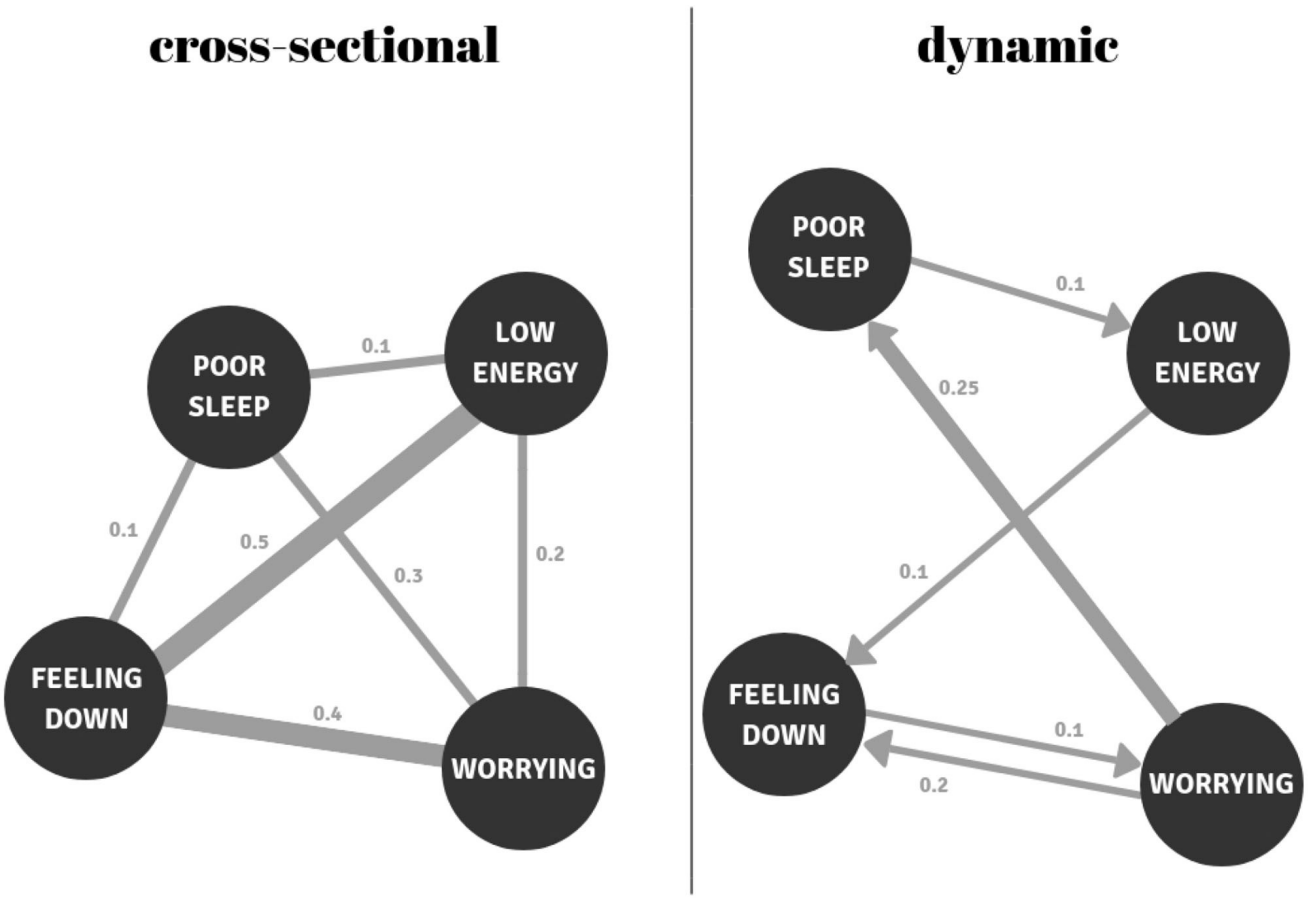
Examples of a cross-sectional and a temporal (dynamic) network. In the cross-sectional network, *feeling down* is the most central node (0.1 + 0.4 + 0.5 = 1.0); it has the strongest connections with the other nodes. In the temporal network, *worry* has the highest outgoing centrality; this node most strongly predicts other nodes (0.25 + 0.2 = 0.45). The node with the greatest incoming centrality is *feeling down* (0.1 + 0.2 = 0.3), because this node is most strongly predicted by other nodes.

Distinguishing between these four methodological approaches is relevant as results of these varying network approaches should be interpreted differently (9–11). Most cross-sectional network studies utilize data from symptom questionnaires to estimate between-person correlations of symptoms at one point in time for a given group of individuals. The associations between variables in such a network demonstrate, at the group level, the probability that these variables tend to occur together (controlling for all other associations). If, for example, those who worry more than others also suffer from sad moods more than others, then the connection between these symptoms will be stronger in such a network. In contrast, networks utilizing time-series data are typically based on the within-person dynamic associations among momentary affective states. Whereas most dynamic networks are based on temporal associations, some studies have also examined the contemporaneous (concurrent) associations. Connections in dynamic networks based on temporal associations show how changes relative to a person's average in one variable follow changes in the other variables within that person. If, for example, individuals start to worry more than usual every time they feel sadder than they usually do, these symptoms will be more strongly connected in the network (see Figure 2). It is important to note here, however, that temporality does not imply causality; temporal associations could also be the result of an unknown third variable. In order to construct dynamic networks, time-series analyses techniques, such as (multilevel) vector auto regression (VAR) analyses (12, 13), are often used. Thus, whereas cross-sectional models mainly express something about the coexistence of different variables at one moment in time at the group-level, dynamic network models say something about how these variables relate to each other *over* time, within individuals.

Unfortunately, when evaluating the meaning of findings brought forward by network studies, network methodologies are often not clearly separated. This complicates our understanding of the meaning of these findings for the network theory. Furthermore, overviews of findings of empirical network studies in individuals suffering from depression are lacking. Two recent reviews have provided a first overview of the network literature on psychopathology in general (3, 14). Such systematic reviews are important because they advance our understanding of how the network theory has, thus far, been operationalized. However, both reviews have not explicitly summarized and compared findings between the different methodological approaches. Furthermore, as these reviews did not solely focus on network studies in depression, a comprehensive evaluation and discussion thereof was beyond their scope. Therefore, we aimed to address this gap in the literature by providing a clear and in-depth overview of the findings of network studies in depression while distinguishing between the aforementioned methodological approaches. Subsequently, we discuss the current status of network research and challenges for both future research and application in clinical practice.

## Methods

We conducted a systematic search in PUBMED and PsycINFO to identify empirical studies that have applied network analysis to investigate (risk of) depression. We searched for papers published before October 2020, in which abstracts included the terms; (i) “depression or depressive or MDD or major depression” or “psychopathology,” combined with one of the following terms “network” or “impulse response” or “vector autoregression” or “VAR” or “qgraph,” or “network approach,” or “network intervention” and (ii) “affect” or “mental states” or “emotion” or “experience sampling” or “momentary assessment” or “depressive symptoms” or “network analysis.” Papers with the terms “fMRI” or “connectomics” or “functional connectivity” or “mouse” or “rat” or “social network” were excluded.

So far, network studies have primarily focused on investigating the comorbidity of depression with other mental disorders, node centrality, and network connectivity. Therefore, papers had to meet the following inclusion criteria: (i) the paper reported empirical results on a network of symptoms or affect states, with at least three nodes pertaining to depressive constructs; (ii) the sample population was selected based on their experience of depressive symptoms in the past, present, or future, and (iii) the paper included results on comorbidity, node centrality and/or network connectivity. As a consequence of this focus on depression, some well-cited network papers fell out of the final selection (2, 15, 16). Furthermore, given that our narrative review focuses on the relationships between symptoms and/or affect states, and the inclusion of other nodes might confound estimates of comorbidity, centrality or connectivity, we excluded papers that included contextual factors as nodes such as treatment, genes, coping strategies, or activities (17–19).

## Results

Our search resulted in 56 network papers spanning the years 2014–2020. Table 1 provides an overview of studies and the methodological approach used to construct networks. Most of these studies used network nodes representing macro-level depressive symptoms (1), assessed with retrospective measures (assessing symptoms of the past week or weeks) via diagnostic interviews or questionnaires (*n* = 45 see Figure 1). Of these studies at the macro-level, most (*N* = 40) constructed cross-sectional networks, although there were also five dynamic studies at the macro-level. Eleven studies used momentary affective states as nodes in the network. These states were assessed with frequent questions, often multiple times a day, using the experience sampling methodology (ESM). With ESM, participants indicate their affect states in that particular moment, such as sadness, irritation, or cheerfulness. We refer to this as network research at the “micro-level” because the examined processes occur on a much smaller time scale (8). Of the eleven micro-level studies, the majority examined dynamic temporal associations (*N* = 8). One study compared three networks: one based on contemporaneous associations, one based on temporal connections, and one based on cross-sectional micro-level data. Another study also constructed both a temporal as well as a contemporaneous network. The final micro-level study solely examined contemporaneous associations in two separately estimated networks. In the following paragraphs, we will synthesize the results of the network studies regarding comorbidity, centrality, and connectivity, while distinguishing between the different methodological approaches.

**Table 1:** Overview of network studies on depression (*N* = 56).

Comorbidity^*^ **(***N*** = 23)**					
**Authors, year (reference)**	***N***	**Time-scale**	**Cross/dyn**	**Population**	**Bridge symptoms**
Afzali et al., 2017 (20)	909	Macro	Cross	PTSD and depression screening	Sense of foreshortened future, guilt, sadness
An et al., 2019 (21)	776	Macro	Cross	MDD diagnosis and high/low anxiety (BAI ≥ 16 Or BAI <16)	No bridge symptoms identified
Beard et al., 2016 (22)	742	Macro	Cross	MDD, bipolar disorder, anxiety disorder, personality disorder and/or psychotic disorder diagnosis	No bridge symptoms identified
Bekhuis et al., 2016 (23)	2,704	Macro	Cross	MDD or GAD diagnosis now or in remission and healthy controls	Anxiety, fatigue, psychomotor agitation
Curtiss and Klemanski, 2016 (24)	111	Macro	Cross	MDD or GAD diagnosis	No bridge symptoms identified
de Haan et al., 2020 (25)	2,313	Macro	Cross	PTSD screening and depressive symptoms	Bridge estimates too unstable
de la Torre-Luque and Essau, 2019 (26)	1,494	Macro	Cross	MDD and social phobia diagnosis	No bridge symptoms identified
Djelantik et al., 2020 (27)	458	Macro	Cross	PGD, PTSD or MDD symptoms	No bridge symptoms identified
Garabiles et al., 2019 (28)	355	Macro	Cross	PHQ-9 ≥ 6 and GAD-7 ≥ 7	Fatigue, sadness, anhedonia
Heeren et al., 2018 (29)	174	Macro	Cross	Social anxiety disorder diagnosis and (some) MDD diagnosis	Avoidance, fear of working while observed, suicidal ideation, anhedonia
Jones et al., 2018 (30)	87	Macro	Cross	OCD diagnosis and (some) MDD diagnosis	Obsessional problems, concentration, guilt, and sadness
Lazarov et al., 2020 (31)	1,795	Macro	Cross	PTSD diagnosis and depression screening	Sleep, sadness, tension, avoidance, upset due to trauma reminders
Levinson et al., 2017 (32)	196	Macro	Cross	Bulimia Nervosa and (some) MDD or anxiety diagnosis	No bridge symptoms identified
Lorimer et al., 2020 (33)	867	Macro	Cross	Relapse: PHQ-9 ≥ 10 and GAD-7 ≥ 8 Remission: PHQ-9 ≤ 10 and GAD-7 ≤ 8	No bridge symptoms identified
McNally et al., 2017 (34)	408	Macro	Cross	OCD diagnosis and (some) MDD diagnosis	No bridge symptoms identified
Park and Kim, 2020 (35)	223	Macro	Cross	MDD diagnosis and anxiety symptoms	No bridge symptoms identified
Rogers et al., 2019 (36)	167	Macro	Cross	MDD, substance use, PTSD or bipolar disorder diagnosis	No bridge symptoms identified
Shim et al., 2020 (37)	907	Macro	Cross	MDD and AUD screening	Men: sadness, suicidal ideation, attempt
					Women: worthlessness, suicidal ideation
Tundo et al., 2020 (38)	241	Macro	Cross	MDD or bipolar disorder diagnosis and anxiety symptoms	No bridge symptoms identified
van Heijst et al., 2020 (39)	618	Macro	Cross	MDD, dysthymia or depressive disorder not otherwise specified diagnosis and autism symptoms	No bridge symptoms identified
Wang et al., 2020 (40)	2,542	Macro	Cross	PHQ-9 ≥ 5 and GAD-7 ≥ 5	During outbreak^**^: psychomotor symptoms
					After peak phase: irritability, energy loss
Kaiser and Laireiter, 2018 (41)	10	Macro	Dyn-C	MDD, social anxiety disorder, or GAD or a combination	Large individual differences in bridge symptoms
Groen et al., 2020 (42)	220	Micro	Dyn-T	MDD and anxiety disorder	No evidence for bridge symptoms
			**Centrality** **(***N*** = 30)**		
**Authors, year (reference)**	**N**	**Time-scale**	**Cross/dyn**	**Population**	**Most central in network**
Berlim et al., 2020 (43)	151	Macro	Cross	MDD diagnosis	Before AD: fatigue, cognitive disturbance, suicidality
					After AD: sadness, psychomotor disturbance, cognitive disturbance
Bos et al., 2018 (44)	178	Macro	Cross	MDD diagnosis	Before AD: energy loss, concentration, worthlessness
					After AD: anhedonia, worthlessness
Boschloo et al., 2016 (45)	501	Macro	Cross	Future MDD diagnosis	Energy loss, concentration, anhedonia
Carney et al., 2018 (46)	125	Macro	Cross	MDD diagnosis	In remission: energy loss, hypersomnia, concentration
					Not in remission: fatigue, energy loss, hypersomnia
de la Torre-Luque et al., 2020 (47)	427	Macro	Cross	MDD diagnosis	Concentration, energy loss, slow thinking
Fried et al., 2016 (48)	3,463	Macro	Cross	MDD diagnosis	Energy loss, anhedonia, appetite
Hakulinen et al., 2020 (49)	595	Macro	Cross	MDD diagnosis	Anhedonia, sadness, energy loss
Kendler et al., 2018 (50)	5,952	Macro	Cross	MDD diagnosis	Psychomotor skills, hopelessness, reduced self-confidence
Madhoo and Levine, 2016 (51)	2,876	Macro	Cross	MDD diagnosis	Before AD: hypersomnia
					After AD: sadness
Murri et al., 2018 (52)	8,557	Macro	Cross	EURO-D ≥ 1	Suicidality, anhedonia, sadness
Park et al., 2020 (53)	1,174	Macro	Cross	MDD diagnosis	Sadness, fatigue, anhedonia
Santos et al., 2017 (54)	515	Macro	Cross	BDI ≥ 10 and healthy controls	Sadness, joy
Santos et al., 2018 (55)	306	Macro	Cross	CES-D ≥ 16	Feeling unwanted, concentration
Semino et al., 2017 (56)	264	Macro	Cross	GDS > 5	Full of energy, hopeless, happy
van Borkulo et al., 2014 (57)	1,108	Macro	Cross	MDD diagnosis now or in remission and healthy controls	Sadness, energy loss, self-criticism
van Borkulo et al., 2015 (58)	515	Macro	Cross	MDD diagnosis	Anhedonia, energy loss, concentration
van Loo et al., 2018 (18)	5,784	Macro	Cross	MDD diagnosis	Psychomotor skills, hopelessness, decreased self-confidence
Bringmann et al., 2015 (59)	182	Macro	Dyn-T	MDD diagnosis	Out: suicidality and anhedonia
					In: indecision and anhedonia
Groen et al., 2019 (60)	60	Macro	Dyn-T	MDD diagnosis	Out: worrying and energy loss
					In: feeling trapped and energy loss
Komulainen et al., 2020 (61)	3,559	Macro	Dyn-T	MDD diagnosis	AD Out: Suicidality, work/activity difficulties, weight loss In: Insight, suicidality, work/activity difficulties
					Placebo Out: Insight, suicidality, retardation In: Insight, retardation, genital symptoms
Savelieva et al., 2020 (62)	72,971	Macro	Dyn-T	EURO-D≥1	Out: suicidality, fatigue, sadness
					In: suicidality, anhedonia, sadness
Bos et al., 2017 (9)	104	Micro	Cross	MDD diagnosis	Worry and self-doubt
Bos et al., 2017 (9)	104	Micro	Dyn-C	MDD diagnosis	Sadness and restlessness
Bos et al., 2017 (9)	104	Micro	Dyn-T	MDD diagnosis	Out: cheerful | In: sadness
David et al., 2018 (63)	1	Micro	Dyn-T	MDD diagnosis	Out: tension | In: concentration
de Vos et al., 2017 (64)	54	Micro	Dyn-T	MDD diagnosis	Out: cheerful | In: tense
Fisher et al., 2017 (65)	1	Micro	Dyn-C	MDD diagnosis	Hopeless and guilty
Fisher et al., 2017 (65)	1	Micro	Dyn-T	MDD diagnosis	Out: positivity | In: content
Wichers et al., 2016 (66)	1	Micro	Dyn-T	MDD diagnosis in remission	Positive affect
Wigman et al., 2015 (67)	129	Micro	Dyn-T	MDD diagnosis	Out: cheerful | In: content
			**Connectivity** **(***N*** = 17)**		
**Authors, year (reference)**	***N***	**Time-scale**	**Cross/dyn**	**Population**	**Hypothesis connectivity**
Baez and Heller, 2020 (68)	3,184	Macro	Cross	MDD diagnosis	Higher connectivity for younger ages of onset
Berlim et al., 2020 (43)	151	Macro	Cross	MDD diagnosis	No support
Bos et al., 2018 (44)	178	Macro	Cross	MDD diagnosis	No support
Hakulinen et al., 2020 (49)	6,593	Macro	Cross	MDD diagnosis and healthy controls	No support
Madhoo and Levine, 2016 (51)	2,876	Macro	Cross	MDD diagnosis	Supports hypothesis
Santos et al., 2017 (54)	515	Macro	Cross	≥10 BDI and healthy controls	Supports hypothesis
Schweren et al., 2018 (69)	465	Macro	Cross	MDD diagnosis	No support
van Borkulo et al., 2015 (58)	515	Macro	Cross	MDD diagnosis	Supports hypothesis
Groen et al., 2019 (60)	60	Macro	Dyn-T	MDD diagnosis	No support
de Vos et al., 2017 (64)	54	Micro	Dyn-T	MDD diagnosis	Supports hypothesis depending on method
Pe et al., 2015 (70)	106	Micro	Dyn-T	MDD diagnosis	Supports hypothesis
Snippe et al., 2017 (71)	169	Micro	Dyn-T	MDD diagnosis	No support
van de Leemput et al., 2014 (72)	93	Micro	Dyn-C	MDD diagnosis	No support
van de Leemput et al., 2014 (72)	621	Micro	Dyn-C	Future increase in SCL-90 depressive symptoms	Supports hypothesis
Wichers et al., 2016 (66)	1	Micro	Dyn-T	MDD diagnosis in remission	Supports hypothesis
Wichers et al., 2020 (73)	6	Micro	Dyn-T	MDD diagnosis in remission	Supports hypothesis
Wigman et al., 2015 (67)	129	Micro	Dyn-T	MDD diagnosis	Supports hypothesis

*Overviews are given for the studies on comorbidity (upper panel), centrality (middle panel), and connectivity (lower panel), respectively. Findings of studies that have constructed multiple networks are given separate rows to distinguish their findings*.

*“And” denotes populations consisting of individuals with both diagnoses or symptom types; “Or” indicates populations consisting of individuals with either one of the diagnoses or symptom types*.

* *For the category comorbidity, inclusion criteria were broadened to also include studies with additional participants who experience other sorts of psychiatric symptoms than depressive symptoms (only)*.

** *Refers to the COVID-19 outbreak*.

*AD, use of antidepressants; AUD, alcohol use disorder; BAI, Beck Anxiety Inventory; BDI, Beck Depression Inventory; CES-D, Center for Epidemiological Studies Depression; Cross, cross sectional associations; Dyn-C, dynamic contemporaneous associations; Dyn-T, dynamic temporal associations; GAD, generalized anxiety disorder; GDS, Geriatric Depression Scale; In, incoming centrality; MADRS, Montgomery-Åsberg Depression Rating Scale; OCD, obsessive compulsive disorder; PGD, prolonged grief disorder, PHQ-9, Patient Health Questionnaire; PTSD, posttraumatic stress disorder; Out, outgoing centrality*.

### Comorbidity

Cramer and colleagues (2) were the first to construct a cross-sectional network with clinical symptoms as nodes (macro-level) to map comorbidity of psychiatric symptoms of depression and generalized anxiety disorder (GAD). Although conducted in a general population sample, the study convincingly demonstrated that symptoms attributed to one diagnostic label (e.g., depression) often co-occur with symptoms associated with differing diagnoses (e.g., GAD). Subsequent studies have since then investigated the comorbidity of depression with other forms of psychopathology. With two exceptions, all studies (*N* = 21) were cross-sectional network studies at the macro-level. Depressive symptoms were most often investigated alongside symptoms of posttraumatic stress disorder (PTSD) and anxiety disorders such as GAD. Other forms of psychopathology that have also been explored include obsessive-compulsive disorder (OCD), prolonged grief disorder, autism, alcohol use disorder (AUD), and somatic symptomatology (see the heading “Comorbidity” in Table 1 for an overview of these studies). Together, these studies confirm that symptoms of depression often co-occur with symptoms of other disorders, which strengthens the conceptualization of psychopathology as transcending boundaries of diagnostic categories (74).

Relatively recently, studies have started to identify so-called “bridge symptoms.” Originally, a bridge symptom was described as a symptom shared by two syndromic clusters (e.g., sleep disturbance in MDD and GAD) (2). However, this definition was later adapted to signify a symptom of one syndromic cluster that has strong connections to symptoms of another cluster (75). Symptoms with high bridge centrality have the strongest connections to nodes of symptoms belonging to another cluster (75, 76). Based on the network theory of psychopathology, it can be hypothesized that comorbidity arises because a bridge symptom of one disorder (e.g., PTSD) also activates symptoms of the other disorder (e.g., depression). Thereafter, activation of symptoms can further expand within the cluster of depression symptoms. Although difficult to directly test this hypothesis, several studies have attempted to identify such bridge symptoms. Notably, the comparison of these studies is complicated because they have operationalized bridge symptoms in different ways: some followed the original operationalization as proposed by Jones and colleagues (2019 preprint, publication in 2021), whereas others relied on visual inspection or other more general centrality measures. For clarity, we will only discuss studies here that have followed the original operationalization, because this stays closest to the original bridge symptoms hypothesis based on network theory (75, 76).

In macro-level studies (*N* = 8), depressive symptoms that were most often identified as a bridge between depression and other disorders were *sadness*, followed by *loss of interest* and/or *pleasure* (anhedonia), *energy loss* or *fatigue*, and *guilt*. It is notable that only two dynamic network studies have been conducted on identifying bridge symptoms between symptom clusters. Such dynamic network studies could further illuminate whether within-person connections between symptoms of different diagnoses might help to explain comorbidity. A first dynamic network study at the macro-level examined the contemporaneous associations among daily reported symptoms for 10 patients separately and reported large individual differences in identified bridge symptoms (41). The second dynamic network study examined temporal associations at the micro-level in patients diagnosed with depression and anxiety disorder. No evidence for bridge symptoms in overlapping momentary states (*irritated* and *worrying*) was found (42). Thus, methodological approaches regarding bridge symptoms have so far yielded inconsistent findings. Further studies investigating bridge symptoms are needed to further validate the bridge symptom hypothesis within persons.

### Centrality

Research has further focused on the centrality of nodes in networks. Centrality measures have been suggested to indicate how influential nodes are in transmitting information to other nodes within the network (77). Most studies have focused on three centrality measures: strength, betweenness, and closeness. However, the latter two have been argued to be unsuitable as measures of node importance in psychological networks (78). Strength centrality, on the other hand, is easy to interpret and is therefore used most often. Strength centrality reflects the weight of connections between a certain node and all other nodes in the network. In cross-sectional networks, this is calculated by summing the absolute weights of all connections of that node. In temporal networks, a distinction can also be made between outgoing and incoming connections of a node (see Figure 2 for further explanation).

Again, most network studies investigated centrality in cross-sectional networks at the macro-level (*N* = 17, see the heading “Centrality” in Table 1), with depressive symptoms as nodes in the network. Four symptoms were most often identified as highly central symptoms in the network, namely: *energy loss* (or *fatigue*), *anhedonia* (loss of pleasure or interest), *depressed mood (*or sadness), and *concentration problems*. One of three studies at the micro-level studying contemporaneous associations also indicated *sadness* as one of the most central symptoms in the networks. As such, it seems that *sadness* is often associated with other symptoms of depression, both at weekly and momentary levels. There are multiple interpretations of this result. *Sadness* and *anhedonia* may be central because they trigger other symptoms of depression. However, another explanation is that *sadness* and *anhedonia* are considered the core symptoms of depression and therefore necessary to be able to receive the diagnosis. They may therefore have the greatest chance of frequently coexisting with other symptoms. This speculation is plausible given that many cross-sectional studies included individuals who met the clinical diagnosis of depression, for whom by definition *sadness* or *anhedonia* should be present. A similar explanation may be applicable for the high centrality of *energy loss* and *concentration problems* in depressive symptom networks.

In dynamic networks based on temporal associations we can distinguish between incoming and outgoing centrality. That is, whether a symptom predicts other symptoms at a later time (outgoing) or is itself predicted by other symptoms at an earlier moment in time (incoming). In particular, symptoms with high outgoing centrality might be interesting in the context of possible interventions within a network structure. This would be the case when one would assume that a node with many outgoing connections strongly influences the rest of the network. Comparing dynamic network studies to cross-sectional ones, we see both similarities and differences. Four studies (see Table 1) looked at the centrality in temporal networks of macro-level depressive symptoms assessed both daily and weekly. In these studies, *suicidality* often ranked among the symptoms with the highest outgoing centrality, followed by *energy loss*, similar to cross-sectional networks. In terms of incoming connections, these studies found that *suicidality* and *anhedonia* were the most central. Here, again, two of the four symptoms correspond to the most central symptoms seen in the cross-sectional studies. The idea that *sadness* would be central, in the sense that this symptom may have a major role in triggering other symptoms, is not supported in these dynamic macro-level network studies based on temporal associations.

The dynamic studies examining temporal associations at the micro-level consisted of three group-based studies and three single-subject network analyses. The findings of the three group-based studies were all consistent concerning outgoing centrality of momentary affect: all indicated *cheerfulness*, or *positive affect* in general, as the most central node. This means that short-term changes in *cheerfulness* or *positive affect* most often preceded short-term changes in other affective states within persons. One of the single-subject studies even showed that as relapse into depressive symptoms drew closer, *positive affect* became increasingly central in this individual's network (66). The finding that *cheerfulness* and *positive affect* were consistently identified as the most influential nodes is interesting. This could indicate that, for many patients, intervening on this node by increasing positive affect could have the greatest impact on the rest of their affective states. Whether this will also be the case in clinical practice still needs to be tested. This finding is in line with psychological theories, such as the “Broaden-and-Build” hypothesis (79, 80), which postulates that positive emotions play an important role in regulating negative emotions, and protect against the negative effects of stress. This finding also partly corresponds to findings in macro-level studies, which often identified *anhedonia* was often identified as a central symptom. *Anhedonia*, as defined by a lack of interest or pleasure, could be viewed as a macro-level expression of a lack of momentary positive affect in daily life (81). Interestingly, in the dynamic macro-level networks, *anhedonia* was mostly identified as being the symptom most often influenced by other symptoms. For now, we can conclude that *anhedonia* and *positive affect* play an important role in the syndrome depression.

As such, converging evidence from different symptom network approaches is found on the relative importance of *anhedonia* and *positive affect* in networks of depressive individuals. Even still, a critical note is in order. Most studies have not tested the ordering of centrality estimates for statistical significance. This limits our ability to assess whether nodes that are identified as central symptoms actually are significantly more central than others. The findings of our review should be interpreted in light of this limitation. We therefore urge researchers to test for ordering which can be done in cross-sectional statistical designs by bootstrapping (82).

### Connectivity

The third topic that has been investigated in network studies is connectivity (also known as density). Connectivity is calculated by summing the weights of all edges within the network. Network theory postulates that depression develops because symptoms or momentary affective states trigger each other over and over again. Therefore, this theory predicts that greater vulnerability to depression is directly related to stronger connectivity within the network (83).

However, studies have reported mixed evidence for this hypothesis. At the macro-level, eight cross-sectional and one dynamic study were conducted. Of these, three cross-sectional studies reported an association between larger connectivity and more current or future depressive symptoms (51, 54, 58). Another study provided indirect support for the hypothesis by relating higher connectivity to an earlier age of onset of depression (68). However, the results of the other five studies were inconsistent with the connectivity hypothesis. Two studies reported increased connectivity after antidepressant treatment (43, 44) and one study did not find stronger connectivity for depressed patients when compared to healthy controls (49). The final cross-sectional macro study attempting to replicate the results of van Borkulo (58) found no indications of higher connectivity predicting worse future development of depression (69). And, finally, the only dynamic (temporal) study at the macro-level also could not confirm that increased connectivity distinguished a worse course from a more favorable course of depression (60).

Therefore, at the macro-level, support for the hypothesis that stronger network connectivity is associated with increased vulnerability to depression is inconsistent. However, these inconsistent findings may simply be due to study design. Studies that failed to find support for this hypothesis compared networks of depressed individuals to networks of remitted patients or healthy controls. It may be expected that samples of healthy or remitted individuals have larger variability in their extent of depressive symptoms than depressed individuals, who may demonstrate mostly high levels of depressive symptoms. Such so-called floor effects are presumed to reduce the strength of associations in a network, and may therefore result in an opposite pattern than would be expected based on network theory (84). In line with this, studies supporting the connectivity hypothesis compared individuals who could be expected to show similar variability in symptoms, by comparing baseline network characteristics between individuals who would later develop depression or stay healthy. More studies are needed to confirm this notion. Another complicating factor when comparing connectivity levels between groups is Berkson's bias (also termed collider bias). This occurs when one estimates symptom associations in multiple groups (e.g., healthy vs. depressed individuals) that were selected based on sum scores of the same (or a similar) questionnaire (85). Because of this bias, spurious negative associations may be uncovered, thereby biasing estimates of connectivity. It is currently unclear how to deal with Berkson's bias, but it can provide another explanation for the mixed results on the connectivity hypothesis thus far.

Eight studies at the micro-level looked at the association between network connectivity and depression, based on the temporal or contemporaneous associations between momentary affective states. Six of them supported the assumption that higher network connectivity indicates a higher vulnerability for depression. Three studies found that depressed patients had a higher network connectivity than healthy controls (64, 67, 70). Two single-subject studies showed that the network connectivity between momentary affective states increased precisely in the weeks preceding a sharp rise in depressive symptoms (66, 73). This finding strongly supports the idea that connectivity between momentary affective states causally affects the development of depressive symptoms. This is especially due to the close temporal association between the increase in connectivity and the moment of sudden change in symptoms. Lastly, one dynamic network study examining contemporaneous associations at the micro-level showed that higher connectivity predicted future depressive symptoms (72).

Two micro-level studies did not support the connectivity hypothesis of the network theory. The first study examined temporal associations and found that treatment with mindfulness or antidepressants did not decrease network connectivity in depressed patients (71). An alternative explanation, also for some of the negative findings regarding treatment effects at the macro-level, could be that treatment does not address underlying vulnerability reflected by the network structure, but rather the symptoms themselves. Moreover, treatment outcomes may differ for each individual. Such heterogeneity may explain why effects are not visible at the group level (71, 86). The second micro-level study that did not support the connectivity assumption examined contemporaneous associations between momentary states. Higher network connectivity at baseline was found in depressed patients who showed higher declines in symptoms in the following year (72). Although inconsistent with the connectivity hypothesis of the network theory, this finding corresponds to another hypothesis based on complex systems theory. From this complex systems theory it is derived that higher connectivity indicates higher instability of the system. An instable system has a higher likelihood to suddenly shift to an alternative stable state, which can be either better or a worse in nature (72, 87, 88). This means that higher levels of connectivity between symptoms or affective states are hypothesized to occur before sudden transitions to alternative states *in general*. Such a transition to an alternative state could entail a sudden increase in symptom levels, similar as in the network theory, but could also entail a decrease in symptom levels. Thus, it may be that system *stability* is more relevant to network connectivity levels, rather than vulnerability *per se*. This might provide an alternative explanation for the mixed findings regarding the connectivity hypotheses both at the macro- and micro-level.

Altogether, although most macro-level studies did not find support for the connectivity hypothesis, the results from most micro-level studies did support this hypothesis. Results largely support the idea that the continuous dynamics between momentary affective states may play an important role in the development of clinical depressive symptoms. Whether this conclusion holds up awaits findings from future research.

## Discussion

This review focused on three hypotheses based on network theory: comorbidity, centrality, and connectivity. First, regarding comorbidity, macro-level cross-sectional studies supported the hypothesis that depressive symptoms tend to co-occur with symptoms of other disorders, and that bridge symptoms that connect depression with other psychiatric disorders can be identified. Such bridge symptoms were often found in *sadness, anhedonia, energy loss* or *fatigue*, and *guilt*. However, given the lack of micro-level temporal studies on this topic, we do not yet know whether such bridges can also be detected within individuals; and whether momentary states associated with different syndromes indeed follow upon each other over time.

Second, in research on the centrality of network nodes, we see different outcomes for macro- vs. micro-level research on the most influential symptoms or affective states. In macro-level studies, most often identified central symptoms are *energy loss, anhedonia, sadness*, and *concentration problems*. At least partly corresponding to these findings, micro-level networks have consistently identified *positive affect* as having high outgoing centrality, suggesting that changes in positive affect strongly influence the rest of the network. However, the number of micro-level network studies is still small. Moreover, it is unknown whether highly central momentary states at the micro-level actually trigger the future development of symptoms assessable at the macro-level.

Third, regarding network connectivity, we found that symptom networks at the macro-level do not consistently support the network theory's connectivity hypothesis. This means that stronger network connectivity was not associated with higher symptom levels or future depressive symptoms in more than half of the studies. In contrast, most results from micro-level studies, examining associations between momentary affective states, did support the connectivity hypothesis. This mixed support for the connectivity hypothesis for depression and depressive symptoms is in conformity with findings for psychopathology in general (3).

The above review of the existing literature shows that the network theory of psychopathology has yielded several interesting areas for further research. As our review has demonstrated, network studies have used different methodological approaches to network theory and, although findings at least partly overlap, each has yielded different conclusions regarding comorbidity, centrality, and connectivity. In future evaluations of the network literature, findings derived from different methodological approaches should be clearly distinguished from one another. We propose that researchers should distinguish between the network theory of psychopathology and the complementary momentary affective dynamics (MAD) network theory, which we will introduce in the next section. After elaborating on the MAD theory, we will discuss four important points to be considered regarding methodological approaches to both network theories. Finally, we will elaborate on the application of network theory in clinical practice.

### Proposing the Momentary Affect Dynamics (MAD) Network Theory

An important question is at which level network dynamics operate. Or more specifically, whether network dynamics operate at the level of clinical symptoms, momentary affective states, or both, to result in the depressive syndrome. In this review, we have reviewed empirical research conducted at both levels. However, most systemic evaluations of the network literature have not taken this distinction into account (3, 14). One reason for this may be that both methodological approaches are described under the theoretical concept of “the network theory of psychopathology,” even though their focus and assumptions are slightly different. For the purpose of transparency, we will therefore name the theoretical approach on the relevance of network dynamics at the micro-level, as described by Wichers (8), the “*momentary affect dynamics (MAD*)” *network theory* of psychopathology. We will shortly summarize the main similarities and differences of this approach to the traditional macro-level network approach as first described by Cramer and colleagues (2) and expanded upon by Borsboom (1).

Both network theories share the assumption that psychological states causally influence one another and that these dynamics play an important role in the further development of psychopathology. An important difference, however, is the level at which network dynamics are assumed to exert their influence, as referenced before. The two theories focus on a different part of the developmental process of psychopathology. The macro-level network theory focuses on the relationships between depressive symptoms. The MAD theory proposes that dynamics between micro-level momentary affective states are actually the building blocks for the development or maintenance of these macro-level symptoms.

Furthermore, the traditional macro-level network theory describes the process of causal influence between symptoms as a serial process with feedback loops. In other words, symptoms develop, which cause other symptoms to develop, and this process continues until it eventually leads to a mental disorder (1). The MAD network theory does not assume such a serial process *per se*. Rather, it assumes that macro-level network connections result from continuously repeating minor impacts of one momentary affective state onto another (8). Since affect states, such as feeling down, cheerful, or irritated, and their fluctuations are frequent everyday experiences, this assumption makes sense at this level of investigation. For example, within a person, affect state A (feeling down) may often impact affect state B (worrying), which often impacts affect state C (feeling less energetic). The higher the connectivity between these negative momentary affective states, the more these dynamics reinforce one another and draw individuals into cycles of persistent and negative psychological states. It is hypothesized that these persistent psychological states are then eventually experienced as symptoms that can be rated on a traditional psychopathology questionnaire. Note that here, dynamic effects are also likely to occur in parallel and that the whole cycle of dynamic effects does not necessarily need to be precisely timed one after another in order to develop psychopathology. Instead, the MAD network theory assumes that when multiple affect states repeatedly negatively impact many other affect states, the risk of getting stuck in a persistent negative psychological state increases.

In this review, we have seen that findings differ between studies based on the macro-level and micro-level network theory of psychopathology. Now that we have outlined the underlying assumptions of both network theories, at both the macro- and micro-level, it may become clearer that differences in findings are simply due to inherent differences between these approaches. By separating the methodological approaches underlying both theories, and naming them the macro-level network theory and the MAD network theory, we aim to facilitate future systematic evaluations of empirical network research.

### The Importance of Individual Models

So far only a few studies have modeled networks per individual (41, 64, 66, 73). These studies show a large heterogeneity in network structure between individuals. Interestingly, within-person network structures also appeared to change over a period in which vulnerability for depression changed (66, 73). At the group-level, connections in time-series networks reflect the average outcome of within-person effects across the entire group. However, the question is then what a group-level network would tell us about the network structure of each individual person within that group (89, 90). Vicious cycles, for example, arising from the dynamics between certain symptoms or momentary affective states, are assumed to be an important risk factor for psychopathology. For instance, an individual could be in a cycle where poor sleep leads to lower energy, which lowers cheerfulness, which triggers worrying, resulting even poorer sleep (see Figure 2). Models at the group-level, however, cannot offer insight into whether these network connections co-occur within every individual within that group. Indeed, some connections may occur only in certain individuals in the group while other connections occur only in other individuals. More idiographic research is thus needed (91), to verify whether presumed vicious cycles found at a group-level actually exist at an individual level. To conclude, there is still an important gap in the testing of assumptions of the network theory using individual models. This is not only a scientific issue, but also a clinical one, as individual models have the potential to bring novel personalized scientific insights into clinical practice.

### The Importance of Studying Processes of Change Using Networks

All but two publications (66, 73) discussed here estimated network structures during a period in which the predicted network parameters were expected to remain constant. These networks were either modeled before symptoms developed or when the symptoms were already present. The process of change over time, thus during a period in which depressive symptoms increase or decrease, has hardly been mapped. If we want to know how symptoms develop and remit, we will have to focus on this dynamic process. This is achieved by creating movies of networks that allow parameters to change in order to visualize their developments (66). If we can demonstrate that network structures already change prior to a change in symptoms, this would strongly support the hypothesis that network structures expose processes that are important in the development of psychopathology. Essential questions remain as to whether or not network connectivity indeed increases shortly before the start of a depressive episode and whether the network structure changes as expected (e.g., that vicious cycles decrease in strength or disappear prior remission). These kinds of studies will be essential to enhance our understanding of the developmental process of psychopathology. This means we will need to collect the necessary longitudinal time-series data, which studies have shown to be feasible (66, 73), and develop data analytical models that can accommodate change. Importantly, the most commonly used network methods require stationarity, which implies that mean levels of symptoms and the associations under study are time-invariant (92) a difficult assumption in clinical psychology, where interventions and simply the passage of time will likely affect symptoms. Recently, models such as time-varying vector autoregressive models have been developed that can handle non-stationarity (93, 94) and enable us to study change in dynamics over time.

### The Importance of the Selection of Network Nodes

Another point of discussion concerns the choice of nodes that are included in a network. First, when aiming to draw conclusions on whether or not symptoms trigger each other, we assume that network connections are not the result of reasons other than causality. Many studies have used the complete list of items from depressive symptom questionnaires or ESM diary questionnaires to construct networks. For a number of these items, however, it seems likely that a third variable is responsible for the co-occurrence of these symptoms (11, 95). For example, reward dysfunction is a likely latent cause for both loss of appetite and loss of pleasure or reduced interest (11). Therefore, to test hypothesis derived from network theory, both the macro-level theory and the MAD theory, it is desirable to prevent the inclusion of nodes with such a common latent cause in the network. Preferably, future studies devise new questionnaires that are designed for network modeling purposes (3) and only include symptom or affect states that really represent distinct facets of depression. The construction of such new questionnaires could be informed by important depressive constructs identified by patients and caregivers (96). Furthermore, networks should focus on the inclusion of contextual variables as nodes in the network, for example treatment (17, 97, 98), life events (99) or social activities (8). Although not the focus of the present review, depressive symptoms do not develop within a vacuum and are likely to be strongly influenced by context. Unfortunately, in micro-level studies, such variables are often measured with categorical response scales, making it difficult to include them in the network. Future research should develop ways to assess context alongside momentary states, to be able to assess their interplay.

### The Importance of Statistical Choices and Pre-processing Steps

The operationalization of network theory faces several statistical challenges that will need to be resolved, some specific to cross-sectional macro-level networks and others specific to micro-level dynamic networks. First, pertaining to both methodological approaches, several empirical studies compared network connectivity between groups of individuals with and without depressive symptoms. However, this is problematic as floor effects in symptoms or affect states may bias estimates of associations between network nodes (84). Similarly, spurious associations may be uncovered when groups are selected based on sum scores of symptoms that are also used as the network nodes (termed Berkson's bias) (85). Floor effects can be circumvented by comparing network connectivity between individuals with equal levels of depression, but different future outcomes of symptom development (58, 69). Models that could address Berkson's bias are currently under development (100).

Second, estimates of network associations depend on the statistical method used (9, 64, 101). In recent years, a large variety of statistical methods have been developed to model networks. Cross-sectional macro-level networks are mostly based on partial correlations between symptoms, using regularization techniques to identify only significant associations (102). Other methods do exist however; for example, relative importance networks or directed acyclic graphs. It is yet unclear how results from these methods relate to one another (103). Since cross-sectional networks have received strong criticisms regarding their stability and generalizability (104, 105), many studies have since then increased their sample size and started to report measures of stability, refraining from reporting network estimates when they can be considered unstable (82, 106). Temporal networks, however, can be estimated on the basis of several techniques, such as multilevel VAR (13), the sparse VAR technique (12), impulse response functions (107), or the graphical VAR model (108). The choice of statistical method can greatly influence the results and thus the conclusions of a study. This is demonstrated by the study of de Vos and colleagues (64), who showed that multilevel VAR and sparse VAR techniques resulted in conflicting results regarding network connectivity in the same data. This inconsistency in statistical models hampers our understanding of the results of networks.

A third challenge is specific to micro-level MAD networks and pertains to the preprocessing steps to be carried out on the data. This occurs even before one has selected the statistical method of choice. These steps may seem trivial, and are often unfortunately left out in publications, but can greatly affect the conclusions drawn from network results (64, 101, 109). Examples of such preprocessing steps are decisions on person-mean centering and the removal of time trends. Others concern decisions regarding whether or not to impute missing values and apply regularization techniques for model optimization (110). To date, there is no gold standard for preprocessing decisions because optimal choices can differ per study design. Further, we simply do not yet know which choices result in network coefficients that adequately reflect psychopathological vulnerability. We therefore urge researchers to be transparent in their publications regarding preprocessing steps and analytical models, and their rationale. This will enable us to better understand conflicting or replicated results. Furthermore, systematic empirical research is needed as this may reveal what type of models and statistical choices yield networks that expose information with true clinical value.

## Where do We Stand in Terms of the Application of Networks in Clinical Practice?

There is rightly a lot of enthusiasm about the network approach in psychopathology among mental health professionals (5, 6). Network theory could be an interesting clinical application in several ways. First, in clinical practice the intuitive idea prevails that the network theory is in line with how psychopathology is expressed, and that these networks can important processes contributing to the development of psychopathology. It is thus a natural fit to clinical frameworks (5). A second advantage of network models is that, hypothetically, they can identify how relevant contextual factors, such as physical activity or social behavior, influence a patient's well-being. Individual networks could also expose the presence of certain vicious cycles of psychological states and behaviors, as has been demonstrated in a patient with psychosis (4), and in a patient with a panic disorder (7). A final advantage lies in the potential of increased network connectivity to alert patients and clinicians of symptom relapse in the near future (66). Applications that use real-time monitoring and detection can thus be envisioned to apply these novel insights into clinical practice (73). In this way, the network approach could help patients and their caregivers to intervene more quickly and in a more focused manner. This is the promise of the network approach for clinical practice.

However, it is apparent from our review that network research is still in its infancy. The empirical testing of network theory has barely begun and still has many challenges. As stated above, it is still unclear which statistical network models can best inform clinical issues and how. Given that the choice of statistical models and preprocessing steps in data analyses can determine the conclusions drawn from networks, this is not a trivial question. Thus, at present, it is not yet possible to guarantee that individual network models based on the data of patients are valid and trustworthy. An illustrative example in this regard is the suggestion that highly central symptoms or momentary states represent good initial treatment targets. However, the use of centrality estimates in psychological networks has been criticized, since the flow process in psychopathology may be radically different from flow process in other types of networks from which it stems [e.g., social networks or virus infections; (78)]. It is therefore questionable whether centrality can be equated with influence, and whether it is indeed informative for intervention targets. Likewise, it is also highly difficult to empirically test whether intervening on a central symptom does directly improve the rest of the system (111). Such hypotheses will need to be empirically tested before they can be implemented in treatment.

Nonetheless, several studies have conducted proof-of-principle experiments to examine if networks could inform diagnostics and treatment (7), monitor the development of symptoms (4) and in early detection for the risk for depressive relapses (66). These applications might be considered successful in the sense that they facilitated the dialogue between patient and therapist, increased motivation for trying out different treatment techniques and improved the self-management of the patient (5, 6, 112). Therefore, despite the fact that the validity of networks is still under discussion, the network approach could be valuable in improving the patient-therapist alliance and encouraging more active involvement of patients in their treatment processes. However, the danger of current applications of network feedback in clinical practice is that patients may get the false impression that these personalized networks provide scientifically validated information, which cannot yet be guaranteed (113). It is therefore important that research clarifies, as quickly as possible, under what conditions networks make proper predictions about vulnerability to psychopathology and result in useful patient-specific knowledge on the best intervention targets. Furthermore, future studies will have to demonstrate the added value of using individual network models in treatment above and beyond existing evidence-based treatment protocols.

## Conclusion

The potential of the network theory is large and cannot be denied. It has both scientific and clinical face validity. This justifies intensive scientific explorations into operationalizations of the network theory. In this narrative review, we have outlined the current state of empirical network studies within the field of depression. We made explicit that at least two conceptually different, but complementary, network theories have been investigated: the traditional macro-level network theory of psychopathology focusing on clinical symptoms, and the MAD network theory focusing on affective states at the micro-level. By systematically differentiating findings of these methodological approaches, we have structured the current empirical support for assumptions of the network theory. Importantly, we argue that we need more empirical studies, and careful systematic evaluation of their findings, to conclude whether network studies can be considered to illuminate the development of psychopathology of individual patients, and whether they provide novel and clinically useful information. Future research may focus on individual rather than group models, processes of change, defining relevant network nodes, and systematic testing of the impact of various statistical specifications on network models. These steps will ensure that the network theory is further consolidated as both a research methodology as well as a clinical instrument.

## Author Contributions

MW designed and conceptualized the review, and wrote the first draft of the manuscript. MW and FB reviewed and selected the literature, and analyzed the results. TH assisted in the literature search and revised the article for grammar. FB, ES, and HR critically revised the review. All authors contributed to and have approved the final manuscript.

## Funding

This study was financially supported by the Rob Giel Research Center, the European Research Council (ERC) under the European Union's Horizon 2020 research and innovation program (ERC-CoG-2015; grant no. 681466 to MW), the Netherlands Organisation for Health Research and Development (ZonMw Off Road; project no. 451001029 to ES), the charitable foundation Stichting tot Steun VCVGZ (grant no. 239 to HR), and Innovation Fund “Stichting De Friesland” (grant no. DS81 to HR and MW).

## Conflict of Interest

The authors declare that the research was conducted in the absence of any commercial or financial relationships that could be construed as a potential conflict of interest.

## Publisher's Note

All claims expressed in this article are solely those of the authors and do not necessarily represent those of their affiliated organizations, or those of the publisher, the editors and the reviewers. Any product that may be evaluated in this article, or claim that may be made by its manufacturer, is not guaranteed or endorsed by the publisher.

## Footnotes

^1^The term “cross-sectional” here refers to data observations collected at the same time point in the research design.
